# Biological Basis of Tree-Ring Formation: A Crash Course

**DOI:** 10.3389/fpls.2016.00734

**Published:** 2016-05-26

**Authors:** Cyrille B. K. Rathgeber, Henri E. Cuny, Patrick Fonti

**Affiliations:** ^1^LERFoB, INRA, AgroParisTech, NancyFrance; ^2^Swiss Federal Institute for Forest, Snow and Landscape ResearchBirmensdorf, Switzerland

**Keywords:** cambial activity, tree growth, tree-ring structure, quantitative wood anatomy, climatic factors, xylem, lignin, cellulose

## Abstract

Wood is of crucial importance for man and biosphere. In this mini review, we present the fundamental processes involved in tree-ring formation and intra-annual dynamics of cambial activity, along with the influences of the environmental factors. During wood formation, new xylem cells produced by the cambium are undergoing profound transformations, passing through successive differentiation stages, which enable them to perform their functions in trees. Xylem cell formation can be divided in five major phases: (1) the division of a cambial mother cell that creates a new cell; (2) the enlargement of this newly formed cell; (3) the deposition of its secondary wall; (4) the lignification of its cell wall; and finally, (5) its programmed cell death. In most regions of the world cambial activity follows a seasonal cycle. At the beginning of the growing season, when temperature increases, the cambium resumes activity, producing new xylem cells. These cells are disposed along radial files, and start their differentiation program according to their birth date, creating typical developmental strips in the forming xylem. The width of these strips smoothly changes along the growing season. Finally, when climatic conditions deteriorate (temperature or water availability in particular), cambial activity stops, soon followed by cell enlargement, and later on by secondary wall deposition. Without a clear understanding of the xylem formation process, it is not possible to comprehend how annual growth rings and typical wood structures are formed, recording normal seasonal variations of the environment as well as extreme climatic events.

## Introduction

Forests are the most widely distributed biomes on earth. They cover one third of the emerged lands, host more than 50% of the world’s biodiversity, and contain more than 60% of the terrestrial carbon pool (Groombridge and Jenkins, 2002). A large part of this carbon is stored in wood, the most abundant biological compound on earth.

The exchanges of carbon dioxide between the forest ecosystems and the atmosphere are crucial processes that influence the balance and the dynamics of the global carbon cycle. Terrestrial plant’s photosynthesis (i.e., atmospheric CO_2_ uptake by the leaves) captures about 120 petagrams of carbon per year (Lal, 2008). Plant’s growth fixes half of this carbon (i.e., 60 petagrams) in form of biomass, while the other half is released back in the atmosphere by autotrophic respiration. Concomitantly, plant’s transpiration releases 40 000 petagrams of water into the atmosphere, influencing global precipitation and heat flux (Seneviratne et al., 2006). Globally, the forest ecosystem photosynthesis draws more carbon from the atmosphere than auto- and hetero-trophic respiration pump into it. So, at the global scale, world’s forests constitute a large and persistent net sink of carbon (Pan et al., 2011). Moreover, the sequestration of carbon into forest’s woody biomass partially counterbalances the current increase of anthropogenic emissions, slowing down climate warming (Bonan, 2008).

Up to now, scientists have considered photosynthesis as the main driver of plant’s growth and have put much effort in better understanding this process. However, another view, which emerged recently, is claiming that, under normal conditions, it is not the source (i.e., photosynthesis) that limit plant’s growth, but the sinks (i.e., the ability of meristems to convert carbon into biomass). In other words, carbon can only be sequestered into wood to the extent cambial activity and environmental conditions permit it (Körner, 2015). However, if after decades of researches on photosynthesis, source activity is very well known and quantified nowadays, cambium functioning is still poorly understood. So, in the context of global warming acceleration, we believe, it is crucial to investigate what is ruling tree-ring formation and wood production, in order to better evaluate how climatic changes are impacting trees, forests, biogeochemical cycles, and ultimately the climate itself.

The process of xylem formation carried out by woody plants is called xylogenesis. The monitoring of the seasonal dynamics of xylogenesis started, more than 50 years ago, with few pioneering works, which aimed to better understand the influence of climate on tree growth, cambium phenology, and wood formation dynamics (Wilson, 1970; Denne and Dodd, 1981). These questions received a renewed attention during the last decade because of the pervasive problem of global changes, leading to a rapid increase in the number of scientific studies involving trees growing in natural or experimental conditions (Griçar et al., 2011). Wood formation monitoring studies are based on repeated (weekly or bi-weekly) cytological observations of the developing xylem all along the growing season. In this mini review, we will briefly present the basics of xylogenesis, along with the current knowledge about the influence of the environmental factors on the cellular processes, the intra-annual dynamics, and the phenology of wood formation, in order to help ecologists to better interpret results from wood formation monitoring studies.

## Wood Structures and Functions

Wood performs four essential functions in trees: (1) supporting and spatially distributing the photosynthetic tissues above ground; (2) conducting the raw sap (i.e., water and nutrients) from the roots up to the leaves; (3) storing carbohydrates, water, and other compounds; and finally (4) protecting the tree from pathogens, by storing and distributing defensive compounds (Kozlowski and Pallardy, 1997).

Wood appeared on earth due to the development of the vascular cambium and the invention of lignin (Rowe and Speck, 2005). The vascular cambium is composed of a thin layer of meristematic cells located between the secondary xylem (i.e., the wood) and the secondary phloem (i.e., the living bark), and forming a continuous envelop all around the stems, branches, and roots of woody plants. The cambium gives rise to xylem inward (i.e., toward the pith), and to phloem outward (i.e., toward the bark).

In gymnosperms (i.e., conifers, or softwoods), xylem is made of a simple and homogeneous tissue, mainly composed of two types of cells: (1) tracheids, which represent more than 90% of the total number of cells, and perform both the mechanical support and the water conduction; and (2) parenchyma cells, which are in charge of the storage and radial transport of various compounds. Tracheids are elongated, spindle-shaped cells of 3–6 mm in length and 6–60 μm in diameter (Sperry et al., 2006). In angiosperms (i.e., hardwoods), xylem is made of a more complex and heterogeneous tissue, composed of several types of cells. Vessels take care of the water conduction, fibers of the mechanical support, and parenchyma cells of the storage. In diffuse-porous trees, vessel dimensions range from 1 to 30 cm in length, and from 15 to 150 μm in diameter; in ring-porous trees, vessel dimensions range from 1 cm to 10 m in length, and from 15 to 300 μm in diameter (Zimmermann, 1983). In both angiosperms and gymnosperms, almost all xylem cells die off at the end of their development to fulfill their functions, only parenchyma cells stay alive for a couple of years.

## Cellular Processes Involved in Wood Formation

**FIGURE 1:**
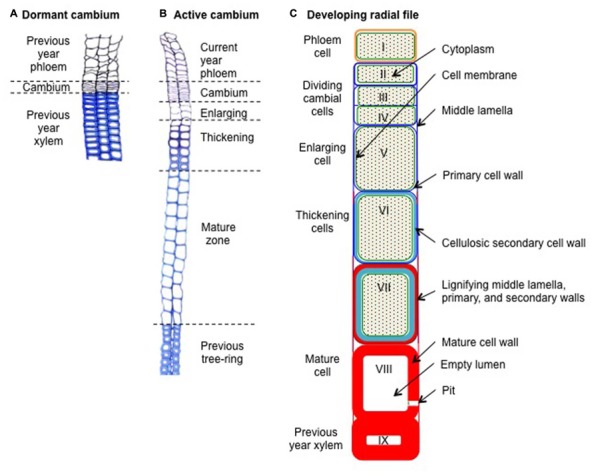
**Schematic cross-sections through resting and developing radial xylem cell files in Scots pine (*Pinus sylvestris* L.). (A)** Dormant cambium during winter composed of a thin strip of 4–6 reinforced cambial cells layers, looking like a “pile of plates,” and embedded between the xylem and the phloem formed during the previous growing season. **(B)** Active cambium and associated developing radial cell files at the beginning of summer. The active cambium, composed of a wide strip of 11–13 cambial cells layers, exhibiting wavy radial cell walls, is embedded between the newly formed phloem and xylem. The developing xylem, which is composed of an enlarging zone (3–4 cell layers), a thickening zone (seven cell layers), and a mature zone (12–13 cell layers), is embedded between the cambium and the previous tree ring. **(C)** Schematic view of a developing xylem radial file. Cell I represents a freshly formed phloem cell of the current year. Cells II–IV represents cambial cells, of which cells III and IV are dividing. Cell V represents an enlarging cell. Cells VI–VII represent thickening cells (note the beginning of the lignification at the corners of cell VI). Finally, cell VIII represent a mature, dead, and fully functional xylem cell (note the disappearance of its cytoplasm). Cell IX represents a latewood cell belonging to the previous tree ring (i.e., formed during the previous growing season). Green backgrounds represent cytoplasm, green lines represent cell membranes, blue lines represent cellulosic cell walls (dark blue for primary walls, light blue for secondary walls), and red areas represent lignified cell walls.

Xylogenesis consists in the production and differentiation of new xylem cells into mature functional wood cells. During their differentiation, xylem cells undergo profound morphological and physiological transformations, which will craft them according to their future functions (Wilson, 1970). The formation of a xylem tracheary element can be divided in five major steps: (1) the periclinal division of a cambial mother cell that creates a new daughter cell; (2) the enlargement of the newly formed xylem cell; (3) the deposition of cellulose and hemi-cellulose to build the secondary cell wall; (4) the impregnation of the cell walls with lignin; and finally, (5) the programmed cell death (**Figure 1**). This sequence is common to both angiosperms and gymnosperms but variations in duration and intensity of the differentiation phases, as well as in molecular components involved, finally result in different cell types and tree-ring structures.

**Cell division** is the elementary process through which the cell number is augmented into a forming tissue. In all the cellular organisms that contain a nucleus (i.e., eukaryotes), dividing cells follow a highly controlled sequence of successive events described as the cell cycle. During this cycle, the meristematic mother cell undergoes several stages of development encompassing cellular growth and DNA synthesis, division of the nucleus, and separation of the cytoplasm, in order to give birth to two daughter cells (Lachaud et al., 1999). The process of cell division is slow in the cambium, with cell cycle duration ranging between 10 and 50 days, depending on tree species, developmental stages, and environmental conditions (Larson, 1994). As a result, the number of cells per developing radial file can only increase by about one cell per day for the most productive trees under the most favorable conditions. Temperature exerts a direct control on cambial cell division, most probably via the polymerization–depolymerisation of the microtubules, a major element of the cell cytoskeleton (Begum et al., 2012). Temperature also influences the division process via hormonal regulation operated by various hormones such as auxins, cytokinins, and gibberellins (Ursache et al., 2013). These phytohormones act in stimulating the synthesis of key proteins: the cyclin-dependent kinases (CDKs), whose enzymatic activity is essential to trigger the start of the cell cycle, and to guarantee its smooth running (Stals and Inze, 2001).

**Cell enlargement** constitutes the first stage of plant cell differentiation. It consists in an irreversible increase of the cell volume (i.e., cell growth) not followed by any cell division. The enlargement of the cell results from (1) the relaxation of the primary cell wall, which (2) creates a passive inlet of water, which (3) is counter-balanced by an active influx of solutes in order to maintain a high turgor pressure (Cosgrove, 2005). The process also requires (4) the biosynthesis and deposition of building material to restore the integrity of the stretched primary cell walls. This process is particularly important for xylem tracheary elements, since their volume is multiplied by 10–100 during this phase. As turgor is the “engine” of cell enlargement, water shortage occasionally affects cell growth. However, under normal conditions, hormonal regulation is the real “driver” of enlargement, determining the final radial diameter of xylem cells. Several phytohormones (e.g., auxins, cytokinins, gibberellins) increase primary cell wall extensibility through different control pathways (Perrot-Rechenmann, 2010).

**Secondary cell walls** are remarkable structures in many plant cells, but they are of particular relevance for woody plants, providing mechanical support, water transport, and biological resistance. Moreover, secondary cell walls represent the major constituent of wood, which is the most abundant pool of terrestrial biomass. Secondary walls are thick (2–10 μm), poorly hydrated (~30%), rigid, and multi-layered. Their principal components are celluloses (40–60% of dry mass), hemicelluloses (10–40%), and lignins (15–35%). Cellulose microfibrils together with hemicellulose form the main load-bearing network, in which lignin is impregnated to form another cross-linked network ensuring hydrophobicity, rigidity, and durability (Zhong and Ye, 2009). Secondary cell walls are commonly composed of three layers: S1, S2, and S3, which present a quite similar composition, but differ in the thickness and orientation of their cellulose microfibrils. The S1 layer is composed of a dense matrix of cellulose and hemicellulose microfibrils. While the microfibrils are oriented transversally in the S1 layer (from 60° to 80° with regard to the cell axis), they change to a longitudinal orientation in the S2 (from 5° to 30° with regard to the cell axis), before coming back to a transversal orientation in the S3 (Plomion et al., 2001). When cell enlargement comes to an end, secondary cell wall formation starts with the deposition, between the membrane and the primary wall, of the S1 layer, soon followed by the S2 and S3 layers. The secondary wall is not covering the whole cell surface, but is absent around the pits. Here the modified primary wall (the pit membrane) allows the passage of water and solutes from one cell to the next —making the upward sap flow from the root tips to the leaves possible. The formation of the secondary cell wall is a complex developmental process supported by the expression of genes activating the biosynthesis, transport, deposition, and assembly of the wall constituents (Zhong and Ye, 2009). A cascade of transcription factors regulates the coordinated expression of all these genes. Phytohormones are also involved in the regulation of secondary cell wall formation, with auxins acting as inhibitors and brassinosteroids as inducers.

**Cell wall lignification** starts at the cell corners, in the primary wall, at about the same time as the deposition of the S1 layer. Then it extends along the middle lamella, and the primary wall, before progressing inward into the secondary wall following its deposition (Donaldson, 2001). Lignin is polymerized directly into the cell wall from oxidized elementary constituents synthesized into the cytosol from phenylalanine to form complex cross-linked phenol polymers. Lignin is then deposited inside the spaces left by the microfibrils, where it forms chemical bonds with hemicelluloses, acting like cement that reinforce and waterproof the cell walls. As a consequence of the timing of the lignification process and the structure of the walls, the proportion of lignin decreases from the most external layers of the cell walls (i.e., the middle lamella and the primary wall) to the most internal ones (i.e., the secondary wall). This well designed structure perfectly fits the physiological functions of the xylem cells. The heavy impregnation of the middle lamella and primary wall by the rigid and hydrophobic lignin molecules, allows xylem tracheary elements to form strong, rigid and self-supporting networks of waterproof “pipes,” while the lighter impregnation of the secondary wall allows tracheary element lumens to keep essential capillarity properties that can support sap ascent. As for the other components of the cell walls, transcription factors play an essential role in the biosynthesis, transport, and deposition of lignin (Zhong and Ye, 2009). Despite the fact that the hormonal control of the lignification process is unclear, it appears that ethylene induces the synthesis of several enzymes involved in lignin biosynthesis. The lignification process is generally described as sensitive to temperature (Donaldson, 2001).

**Programmed cell death** (also called apoptosis) marks the end of xylem cell differentiation and the advent of mature, fully functional, xylem elements (tracheids for gymnosperms, vessels and fibers for angiosperms). It is a highly coordinated and active process of cellular “suicide,” which is widespread in multicellular organisms. However, while most cells perform a specific function until their death, xylem tracheary elements die to become functional. In xylem, only parenchyma cells escape programmed cell death and remain alive for several years. The principal trigger of programmed cell death is a massive influx of calcium ions (CA^2+^) into the vacuole through plasma membrane channels. Death then manifests rapidly (in few minutes or so) as a sudden break-up of the vacuole and the cessation of the cytoplasmic streaming. Moreover, the vacuole break-up releases hydrolases, which attack and degrade the cell organelles and clean the cell content (Bollhöner et al., 2012). After a couple of days, the cell is finally left as an empty space (the lumen) surrounded by a thick wall pierced of pits. In xylem cells, programmed cell death regulation mechanisms appear inextricably linked to those governing secondary cell wall formation. For example, the brassinosteroids that promote secondary wall formation also initiate programmed cell death. In their seminal work, Groover and Jones (1999) proposed a biological mechanism linking together xylem cell apoptosis and secondary wall formation. This mechanism involves the accumulation of a protease in the extracellular matrix during wall material deposition. When the activity of the protease reaches a critical threshold, it triggers the influx of calcium ions, which, in turn, triggers the process of apoptosis. Programmed cell death is an essential step of xylem cell differentiation, allowing mature xylem cells to perform their specific functions in trees. The cell walls (in particular in tracheids and fibers) endow the function of mechanical support to the wood, while the empty cell lumens and the pits (in particular in tracheids and vessels) offer the necessary pathway for water transport into the plant.

## The Seasonal Dynamics of Wood Formation

Cambial activity follows the cycle of the seasons (Denne and Dodd, 1981). In extra-tropical regions, the cambium is dormant during winter and active during summer (Delpierre et al., 2015), while in tropical regions it may rest during the dry season and be active during the wet season (Breitsprecher and Bethel, 1990). Annual growth rings and typical tree-ring structures both result from these periodical changes in cambial activity (Evert, 2006).

During winter, the dormant cambium is composed of a few layers of cells (3–6), presenting thickened primary cell walls (**Figure 1A**). Each spring, when day length increases and temperature rises, cambium resumes activity with the division of mother cells (Prislan et al., 2013). During the growing season, the active cambium is composed of numerous dividing cells (6–18), presenting thin tangential cell walls (**Figure 1B**). A couple of days or weeks after the start of cambial cell divisions, newly created xylem cells appeared in the developing xylem. The enlarging cells still only consist of primary cell walls, but present much wider radial diameters than dividing cells. The appearance of these first enlarging cells marks the onset of xylem radial growth and wood formation. A couple of weeks after their birth, these first cells start to thicken, building their secondary walls. Because secondary walls hold most of the biomass, the appearance of the first thickening cells can be seen as the effective beginning of carbon sequestration into wood. Finally, 1 or 2 months after their birth, differentiating xylem cells reached their final mature state. Mature and fully functional xylem treachery elements are composed of thickened secondary cell walls surrounding empty lumens.

**FIGURE 2:**
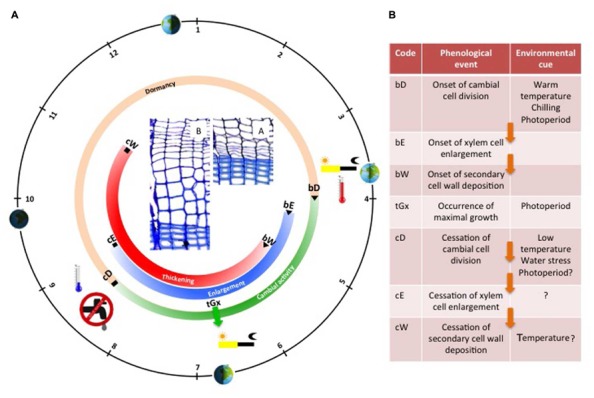
**Schematic representation of the seasonal cycle of cambial activity and tree ring formation in temperate coniferous trees.** The black circle represents the solar calendar; the orange/green circle illustrates the seasonal evolution of cambial activity, while the blue and red circles illustrate the seasonal evolution of wood formation (enlargement and thickening period respectively). Wood formation critical dates are listed in the adjacent table along with their corresponding environmental cues (question marks indicate uncertain roles or missing evidence). Arrows indicate causal relationships between phenological phases. In the center, two cross sections show the cambium and the xylem of a Scot pine tree during winter **(A)**, and spring **(B)**.

During the growing season, new xylem cells, resulting from cambial cell division, are disposed along radial files, and successively undergo the differentiation program phases according to their identity and their place in the “queue” (i.e., the radial file). At the tissue level, the succession in time of cells belonging to the different stages of differentiation is well coordinated between all the radial files, creating a characteristic spatial pattern composed of strip-like developmental zones (**Figure 1B**). Once established, this organization remains rather stable throughout the growing season (Vaganov et al., 2006). Provided that no environmental stress comes into play, cambial activity and xylem radial growth rate generally peak around the summer solstice, when the photoperiod is maximal (**Figure 2**). This period generally marks the transition between earlywood and latewood (Cuny et al., 2014).

At the end of the growing season, in autumn —or even earlier if water or temperature conditions are not favorable anymore— cambial activity stops, soon followed by cell enlargement (flagging the end of stem radial growth). However, the completion of wood formation (marking the end of carbon sequestration) only occurs a couple of months later (Cuny et al., 2015). Indeed, lignification is a slow process constrained by temperature, so the last xylem cells need up to 2 months for ending cell wall maturation and reaching maturity (Cuny and Rathgeber, 2016).

## Conclusion

The cambium and the developing xylem form a complex dynamic system that periodically produces wood according to the cycle of the seasons. Without a clear knowledge of the biological processes at play in each component of this system, it is not possible to understand how xylogenesis responds to environmental conditions, and how it creates typical tree-ring structures endowing specific functions to the wood. Furthermore, taking into account the interactions between the environmental drivers, the physiological state of the trees, and the developmental stage of the forming xylem, is required to comprehend the creation of the typical tree-ring structures during normal seasonal cycles, as well as special anatomical wood features formed under exceptional conditions (e.g., extreme climatic events).

## Author Contributions

CR wrote the manuscript and prepared the figures, with the assistance of HC and PF.

## Conflict of Interest Statement

The authors declare that the research was conducted in the absence of any commercial or financial relationships that could be construed as a potential conflict of interest.
